# Gut microbiome: decision-makers in the microenvironment of colorectal cancer

**DOI:** 10.3389/fcimb.2023.1299977

**Published:** 2023-12-12

**Authors:** Jingrun Han, Biao Zhang, Yongnian Zhang, Tianyi Yin, Yuying Cui, Jinming Liu, Yanfei Yang, Huiyi Song, Dong Shang

**Affiliations:** ^1^ Department of General Surgery, The First Affiliated Hospital of Dalian Medical University, Dalian, China; ^2^ Laboratory of Integrative Medicine, The First Affiliated Hospital of Dalian Medical University, Dalian, China; ^3^ Departments of Gastrointestinal Surgery, The Second Affiliated Hospital of Dalian Medical University, Dalian, China; ^4^ Institute (College) of Integrative Medicine, Dalian Medical University, Dalian, China

**Keywords:** colorectal cancer, gut bacteria, tumor microbial microenvironment, immune escape, therapy

## Abstract

Colorectal cancer (CRC) is a common malignancy of the gastrointestinal tract, accounting for the second most common cause of gastrointestinal tumors. As one of the intestinal barriers, gut bacteria form biofilm, participate in intestinal work, and form the living environment of intestinal cells. Metagenomic next-generation sequencing (mNGS) of the gut bacteria in a large number of CRC patients has been established, enabling specific microbial signatures to be associated with colorectal adenomato-carcinoma. Gut bacteria are involved in both benign precursor lesions (polyps), *in situ* growth and metastasis of CRC. Therefore, the term tumorigenic bacteria was proposed in 2018, such as *Escherichia coli*, *Fusobacterium nucleatum*, *enterotoxigenic Bacteroides fragilis*, etc. Meanwhile, bacteria toxins (such as cytolethal distending toxin (CDT), Colibactin (Clb), *B. fragilis* toxin) affect the tumor microenvironment and promote cancer occurrence and tumor immune escape. It is important to note that there are differences in the bacteria of different types of CRC. In this paper, the role of tumorigenic bacteria in the polyp-cancer transformation and the effects of their secreted toxins on the tumor microenvironment will be discussed, thereby further exploring new ideas for the prevention and treatment of CRC.

## Graphical Abstract

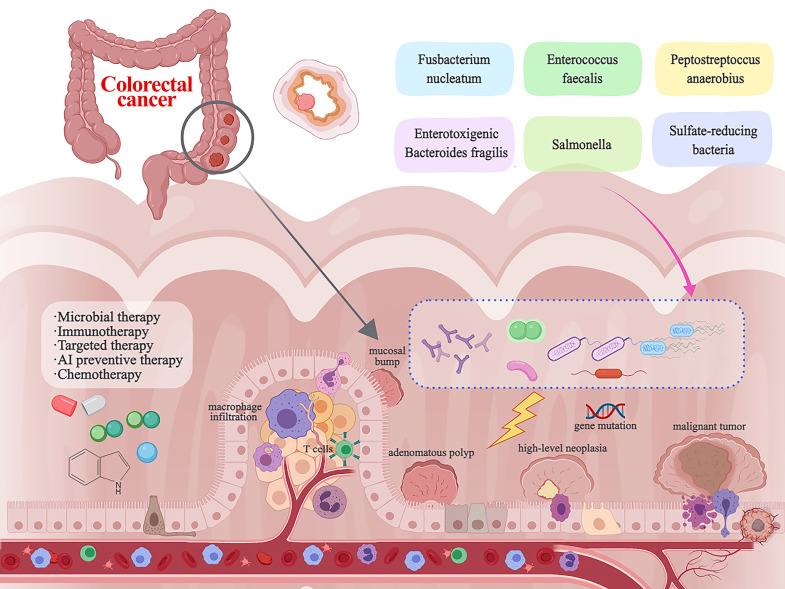

## 1 Introduction

Colorectal cancer (CRC) is a malignant tumor of the colon or rectum that usually originates from mucosal epithelial cells. It is a common type of cancer with high incidence rates worldwide. Several risk factors, such as age, family history, dietary habits, intestinal polyps, and inflammatory bowel disease, are associated with the development of CRC (Center et al., 2009). The intestinal microbiome is a multifaceted ecosystem consisting of a rich array of bacteria, viruses, and fungi. It harbors a vast reservoir of genetic diversity, surpassing that which resides within an individual’s own DNA, making it a profoundly intricate and unique entity. The intricate interplay between bacteria and the host leads to multifaceted impacts of intestinal microbiota and their metabolites on the initiation and progression of CRC, as well as the modulation of the immune microenvironment. Intestinal colonizing bacteria secrete metabolites and enter the blood circulation, thereby affecting important physiological processes such as nutrient absorption, material metabolism, and immune defense (Sun et al., 2023). Moreover, the oncogenic flora promotes the occurrence of CRC by inducing DNA damage in epithelial cells, which in turn promotes the proliferation of bacteria that have a growth advantage in the tumor microenvironment (Tjalsma et al., 2012; Clavenna et al., 2023). The definition of intestinal microbiome is becoming more and more clear, and it is related to countless health conditions. These interactions are now understood to occur locally and throughout the body through changes in the immune system and other mechanisms. The local proximity of intestinal microbiome to the colon led many early researchers to study its effect on CRC, making CRC a frontier for studying the response of microbiome to cancer development, progression and treatment.

## 2 The occurrence and development of CRC

CRC originates from the mucosal epithelial cells in the colorectal mucosa layer. Clinically, CRC is mainly secondary to intestinal polyps and inflammatory bowel disease (Dyson and Rutter, 2012; Wolf et al., 2023). Novel ideas about CRC progression course are that normal mucosa after mucosal bump, small adenomatous polyp, large adenoma, high-level neoplasia, eventually into malignant tumor. The types of polyp tissue prone to cancer include tubular adenoma, villous adenoma, tubular-villous adenoma (mixed adenoma), and serrated adenoma (Knudsen et al., 2023). In a recent investigation, researchers delved into the composition of “mucosal-associated metabolites” in low-grade versus high-grade dysplastic polyps. Notably, they observed an enrichment of the genus *Pelomonas*, a member of the *Proteobacteria phylum*, in the low-grade dysplastic polyps. Conversely, microbiota analyses of high-grade dysplastic adenomas unveiled an elevated presence of the genus *Anaerococcus*, a taxon that has been notably abundant in CRC tissues (Clavenna et al., 2023). In a clinical study of Chinese patients, it was found that *Bifidobacterium bifidum*, *Candida albicans*, and *Saccharomyces cerevisiae* in the feces of CRC patients were more prevalent than those of healthy population (Li X. et al., 2023). In research conducted among individuals diagnosed with familial adenomatous polyposis (FAP), the colonic biofilms were observed to harbor oncogenic bacteria, primarily *Escherichia coli* and *Bacteroides fragilis (* Dejea et al., 2018).

In approximately 85% of colon cancers, the adenomatous polyposis coli (APC) gene, a critical tumor suppressor, undergoes deletion or inactivation (Grivennikov et al., 2012). APC gene is not only associated with FAP, but also plays an important role in the occurrence of CRC. NOTUM retains tumor suppressor activity in APC-ineffective adenomas. However, NOTUM becomes a specific oncogene when it develops into adenocarcinoma with p53 deletion (Tian et al., 2023). Oncogenic microbial communities wield the ability to reshape the entire gut microbiota’s composition, inciting pro-inflammatory reactions and incipient cellular metamorphosis, culminating in carcinogenesis (Yan et al., 2023). Furthermore, oncogenic microbiota catalyze CRC progression through the instigation of DNA damage within the epithelial cells. Epithelial barrier damage may be a consequence of β-catenin activation as well as loss of APC, microbial products drive IL-23/IL-17-mediated tumor growth (Grivennikov et al., 2012).

As early as 2012, the bacterial driver–passenger model was proposed (Tjalsma et al., 2012). Certain driver bacteria, such as *E. faecalis*, produce extracellular superoxide, which causes cellular DNA damage (**Table 1**). In a 16s RNA sequencing discovery, 7 bacterial genera were identified as potential drivers (e.g., *unclassified Pseudomonadaceae* and *Neissenaceae*) and 12 bacterial genera as potential passengers (e.g., *Staphylococcus* and *Veillonella*) (Geng et al., 2014). Some studies have also proposed the “Alpha-bug” model (Sears and Pardoll, 2011; Avril and DePaolo, 2021), *enterotoxigenic Bacteroides fragilis* induces colon tumors in mice (Sears and Pardoll, 2011; Yu and Fang, 2015).

**Table 1:** CRC-associated bacteria.

Strain	Pathogenic metabolites	Mechanism	Reference
*Enterococcus faecalis*	Extracellular Superoxide	DNA damage	(Evans et al., 2004; Tjalsma et al., 2012)
*Escherichia coli*	Polyketide synthetase	Induces single-strand DNA breaks	(Tjalsma et al., 2012)
*Bacill* *us fragilis*	B. fragilis toxin (Metalloproteinase)	Promotes T helper 17 cells to increase expression of interleukin-17 (IL-17) to increase tumorigenesis	(Sears and Pardoll, 2011; Tjalsma et al., 2012)
		Increased intestinal barrier permeability	(Sears and Pardoll, 2011)
		Wnt, NF-κB and Stat3 signal transduction	(Sears and Pardoll, 2011; Yu and Fang, 2015)
*Streptococcus bovis*	*S.bovis bacterial* wall extracted antigens	Inflammation-based sequence of tumor development or dissemination by IL-1, COX-2, and IL-8	(Biarc et al., 2004)

## 3 Gut bacterial products associated with CRC

Bacteria can obtain the ability to penetrate the intestinal mucosal barrier through flagella, pili, and adhesins, as well as adhere to and invade intestinal epithelial cells, produce endotoxin or exotoxin, and then form pathogenicity (Perez-Lopez et al., 2016). Common pathogenic bacteria have been mentioned before and will not be repeated.

A recent study has suggested that an analysis of the microbial community in tumors holds the potential to identify distinct prognostic subtypes of CRC. This classification system delineates three principal subtypes: OCS1, predominantly associated with *Fusobacteria* and oral pathogens; OCS2, characterized by a prevalence of *Firmicutes* and *Bacteroidetes*; and OCS3, featuring an abundance of *Escherichia*, *Pseudomonas* and *Shigella (* Mouradov et al., 2023). OCS1 tumors mostly occur in the right colon and have high pathological grade. In contrast, OCS2 and OCS3 tumors are mostly located in the left colon and rectum with low pathological grade (Mouradov et al., 2023). There was no significant difference in clinical features between OCS2 and OCS3 (Mouradov et al., 2023). It has been found that the expression of Gal‐GalNAc (recognized by Fusobacterium Fap2) may promote the binding of *Fusobacterium* to CRC (Abed et al., 2016). *F. nucleatum* utilizes the non-lectin structure of Clostridium Fap2 to achieve tumor-promoting effects (Alon-Maimon et al., 2022). Additionally, in a pathological context, *F. nucleatum* augments its virulence through the secretion of an amyloid-like adhesin called FadA, utilizing a Fap2-like autotransporter (Meng et al., 2021). In addition, *F. nucleatum* can enhance drug resistance of tumor cells, inhibit neutrophil infiltration, and ultimately change the tumor immune microenvironment (Alon-Maimon et al., 2022; Garcia-Serrano et al., 2023). *F. nucleatum* is involved in tumor initiation or progression before cancer formation, which regulating the tumor immune microenvironment and promoting the proliferation of tumor-infiltrating immune cells (Kostic et al., 2013). *F. nucleatum* pro-inflammatory genes are characterized by upregulation of PTGS2 (Kostic et al., 2013). Nevertheless, certain experiments have revealed that *F. nucleatum* is not an unequivocal instigator of cancer (Nawab et al., 2023). Instead, its carcinogenic potential hinges on the particular dietary context in which it operates.

*E. coli* is involved in the development of CRC through the induction of inflammation and genotoxic host responses by bacteria-derived virulence factors. Some strains of *E. coli* produce a secondary metabolite called colibactin (Clb), and bacteria carrying pks genomic islands have DNA-damaging properties associated with CRC (Dougherty et al., 2023; Harnack et al., 2023). Blocking bacterial adhesion attenuates colibactin-mediated genotoxicity and CRC exacerbations (Jans et al., 2023). Pks+ *E. coli* can opportunistically enter the epithelium and promote existing mucosal damage, while mice colonized with pks+ *E. coli* cannot reestablish functional barriers (Harnack et al., 2023). Grotesquely, it has also been found that about half of colibactin-producing *E. coli* (CoPEC) can encode cytotoxic necrotizing factor-1 (CNF1) which induces CRC in mice by reducing CoPEC (Chat et al., 2023). The influence of microorganisms such as *F. nucleatum*, *E. coli*, *enterotoxigenic B. fragilis*, and *Faecalibacterium prausnitzii* on miRNAs is well-established, and this microbial impact leads to the stimulation of tumor growth and exacerbates inflammatory responses (Xing et al., 2022). Microbiota reprograms mouse intestinal lipid metabolism by suppressing expression of lncRNA Snhg9 in small intestinal epithelial cells (Tian et al., 2023).

*Lostridium sporogenes* is responsible for breaking down tryptophan and secreting the metabolite indole propionic acid (IPA), which has been shown to help strengthen the intestinal barrier and interact with the immune system, then change the biological characteristics of the intestine (Dodd et al., 2017). The gut microbiota metabolizes tryptophan to generate Indole-3-acetic acid (3-IAA), which effectively downregulates the expression of TNF-α. This reduction in TNF-α expression is attributed to the enzymatic conversion of tryptophan, highlighting the microbiota’s significant role in modulating inflammatory responses (Tomii et al., 2023). Furthermore, the metabolization of tryptophan by the bacterial flora results in the production of indole, which exerts regulatory control over mucosal immunity by activating receptors associated with polycyclic aromatic hydrocarbons (Lavelle and Sokol, 2020; Hezaveh et al., 2022). *Bacteroides thetaiotaomicron* inhibits tumor growth by producing short-chain fatty acids (SCFAs) such as propionate (Xu et al., 2023). Elevating the abundance of species such as *Ruminococcaceae*, *Parabacterium*, and *Blautellae* known for their capacity to generate SCFAs, Zearalenone (ZEA) exhibits a notable capacity to effectively suppress the development of colorectal tumors (Leung et al., 2023). The initiation of AhR signaling is triggered by microbiome-derived formate, which subsequently leads to the expansion of Th17 cells and promotes CRC tumor invasion (Ternes et al., 2022).

The occurrence and progression of CRC are influenced by DNA mismatch repair (MMR). In a recent examination of DNA mismatch repair deficiencies (dMMR) versus proficient DNA mismatch repair (pMMR), researchers investigated the impact of microbial-driven metabolic reconfiguration (Hale et al., 2018; Li J. et al., 2023). In the realm of dMMR, a total of 211 distinct species thrived, with noteworthy representatives including *F. nucleatum*, *A. muciniphila* and *O. splanchnicus (* Hsueh et al., 2022; Li J. et al., 2023). In stark contrast, a mere 2 species displayed a deficiency in dMMR, as exemplified by *F. plautii*. Furthermore, the dMMR environment boasted 13 metabolites in abundance, with retinoic acid being a prominent member, while on the opposite end of the spectrum, 77 metabolites experienced a significant depletion in the dMMR context, encompassing lactic acid, succinic acid, and 2,3-dihydroxyvaleric acid (Li J. et al., 2023).

The improved prognosis of colon cancer can be attributed to specific mucosal biota, namely *Faecalibacterium prausnitzii* and *Ruminococcus gnavus*. These microorganisms play a pivotal role by producing metabolites that encompass a spectrum of fatty acid species, including medium chain (MCFAs), long-chain (LCFAs), and very long-chain (VLCFAs) fatty acids, alongside ceramides and lysophospholipids (Alexander et al., 2023).

Similarly, gut bacteria can also produce substances that reverse CRC progression. In a study of female CRC patients, it was found that *Carnobacterium maltaromaticum* was missing (Li Q. et al., 2023). Intestinal colonization of *C. maltaromaticum* is influenced by estrogen and increases the abundance of vitamin D-related metabolites in colon tissue (Li Q. et al., 2023). Remarkably, the progression of CRC has been observed to be exacerbated by alterations in the male gut microbiome (Wang L. et al., 2023). This includes an augmentation in the presence of the pathogenic bacterium *Akkermansia muciniphila* and a reduction in the levels of the beneficial probiotic *Parabacterium kingeri (* Wang L. et al., 2023).

## 4 Gut bacteria regulate the tumor microenvironment

The CRC tumor microenvironment (TME) constitutes a multifaceted and intricate ecosystem, and plays a pivotal role in tumor growth, metastasis, and treatment response. TME comprises a diverse array of cellular components and molecular elements. It encompasses tumor cells, immune cell populations, vascular networks, fibroblasts, intestinal flora and the extracellular matrix (ECM) (Zhang et al., 2023).

It is currently believed that the TME of CRC mainly consists of the intestinal bacteria microenvironment, the inflammatory microenvironment and the hypoxic microenvironment, which work together and coordinate with each other (Wang et al., 2017). This article mainly describes the impact of intestinal bacteria on TME. *Bifidobacterium adolescentis* is a probiotic found in the human intestine. It can inhibit the proliferation of patholgen in the intestine and maintain the homeostasis of the bacterial microenviroment. It has been experimentally confirmed that *B. adolescentis* inhibits tumorigenesis by inducing a new CD143^+^ cancer-associated fibroblasts through Wnt signaling-regulated GAS1 (Chen et al., 2023). In addition, *B. adolescentis* inhibits colorectal carcinogenesis through TLR2 induction of decorin^+^ macrophages (Lin et al., 2023). In AOM/DSS-induced mice, *B. thetaiotaomicron* suppresses tumorigenesis of colitis-associated CRC and MC38 allograft tumors (Xu et al., 2023). Not only in CRC, but other experiments have shown that in melanoma, *Eubacterium rectale* significantly improves the efficacy of anti-PD1 treatment and the overall survival rate of tumor-bearing mice (Liu et al., 2023). *Eubacterium rectale* consumes l-serine to enhance NK cell function and anti-PD1 therapeutic effect, leading to activation of NK cell activity through the FOS/FOSL2 signaling pathway (Liu et al., 2023).

In an *in vitro* study, *F. nucleatum* infection was found to induce a significant increase in the production of neutrophil extracellular traps (NETs) (Kong et al., 2023). This demonstrates that *F. nucleatum*-induced NETs indirectly accelerate malignant tumor growth through angiogenesis and promote tumor metastasis. This is exemplified by cellular migration linked to the process of epithelial-mesenchymal transition (EMT), the breakdown of basement membrane proteins facilitated by matrix metalloproteinases (MMPs), and the entrapment of CRC cells (Kong et al., 2023). In research, exposure of peripheral blood mononuclear cells (PBMCs) to LPS derived from these microorganisms revealed that *F. periodonticum* triggers cytokine synthesis in PBMCs, whereas both *B. fragilis* and *P. asaccharolytica* exerted a suppressive influence (Sulit et al., 2023). In a study of intratumoral bacteria, elevated autophagy induced by *F. nucleatum* led to increased resistance to reactive oxygen species (ROS) in CRC, this resistance was alleviated, ultimately promoting apoptosis in cancer cells, and apoptosis was triggered by intracellular redox imbalance caused by the interaction with BSA-Cu SAN (Wang X. et al., 2023).

## 5 Metastasis and immune escape of CRC cells

Studies have shown that relevant DNA analysis of CRC patients and fecal microorganisms found that KRAS gene mutations have a significant impact on distant metastasis of CRC (Sui et al., 2020). At the same time, in CRC, the abundance of different bacterial groups is also influencing the mutation of KRAS gene, which affects the metastasis and progression of CRC (Sui et al., 2020). Microorganisms such as *Rosella, Paramecium, Post-Rosella, Staphylococcaceae and Bacillariophyta* in the mutant group significantly affected distant metastasis of CRC through KRAS gene mutation, and their prevalence and metastasis were significantly higher than those in the non-mutant group (Liu et al., 2021). Furthermore, butyrate, a prominent component among SCFAs, plays a pivotal role in the metabolic processes of normal colorectal epithelial cells (Yan et al., 2024). Remarkably, a substantial portion of butyrate remains unmetabolized, largely attributed to the fact that colon cells have a Warburg effect pathway (Eslami et al., 2020). Butyrate serves as a potent histone deacetylase (HDAC) inhibitor, influencing the intricate orchestration of tumor cell metabolism, proliferation, and apoptosis (Korsten et al., 2023). Consequently, these multifaceted interactions exert a significant impact on the metastatic potential of CRC (Li et al., 2021). At the same time, it was shown that *F. nucleatum* was found to be highly abundant in CRC and promote CRC metastasis by affecting the miR-1322/CCL20 axis and M2 polarization (Xu et al., 2021). The ALPK1/NF-κB/ICAM1 pathway can be induced by *F. nucleatum*, leading to enhanced adhesion of CRC cells to intestinal endothelial cells, as well as increased infiltration and distant metastasis (Zhang et al., 2022). Additionally, EVADR induction has the potential to facilitate CRC metastasis through YBX1-dependent translation processes (Lu et al., 2022). It has been reported that sustained *F. nucleatum* exposure reduces the diversity of the intestinal microbiota in mice, leading to an imbalance of the intestinal bacteria, and a reorganization of the associated bacteria, which intricately affects colorectal carcinogenesis and progression through the secretion of pro-inflammatory cytokines (Yin et al., 2022).

**Figure 1:**
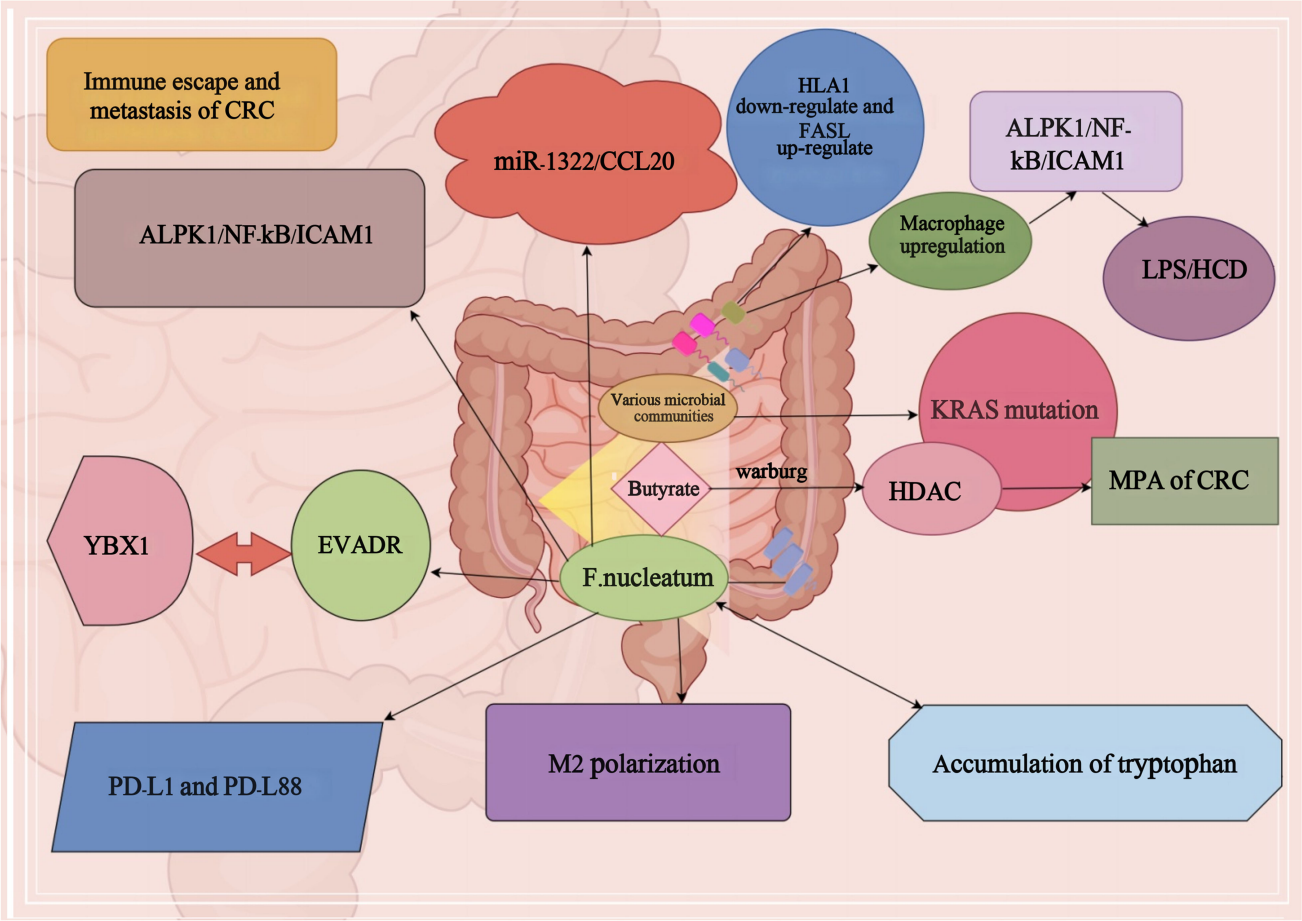
Immune escape and metastasis of colorectal cancer.

*F. nucleatum p*romotes CRC progression and upregulates PD-L1 protein expression in CRC cell lines, thereby promoting immune escape from the tumor (Gao et al., 2023). Furthermore, studies have shown that the accumulation of tryptophan derivatives in the gut promotes the formation of suitable targets for immune escape (Puccetti et al., 2015). Simultaneously, the oncogenic bacteria in the gut, or the metabolites they generate, stimulate the generation of macrophages. The presence of LPS or HCD-induced macrophage infiltration notably triggers the activation of the macrophage-derived CCL5-p65/STAT3-CSN5-PD-L1 signaling pathway, which plays a crucial role in facilitating immune evasion in CRC (Liu et al., 2020). *F. nucleatum* can also lead to tumor subclones with PD-L1 mutations, nonsense-mediated RNA decay in PD-L1 K1fs, and protein degradation in PD-L162 L1S, thereby promoting its immune escape and tumor metastasis (Stein et al., 2021). It has also been shown that metabolites associated with *F. nucleatum* can affect up to 50% of dMMR/high microsatellite instability (MSI-H) advanced cancer patients who progress after PD-1 blockade, leading to a high probability of immune escape (Cohen et al., 2020). *F. nucleatum* has the capacity to promote CRC immune escape by influencing the depletion of human leukocyte antigen class I (HLA-I) (Anderson et al., 2021). In addition, *F. nucleatum* can also help colon cancer evade immune surveillance and immune elimination by influencing Fas expression (O’Connell et al., 2000). Simultaneously, it can bolster the resistance of CRC to the immune system through the upregulation of FasL expression (Zhu et al., 2005). In summary, as mentioned in **Figure 1** CRC immune escape and distant metastasis can be caused by the joint action of intestinal carcinogenic flora and their metabolites.

## 6 Microbiological therapy for CRC

**Figure 2:**
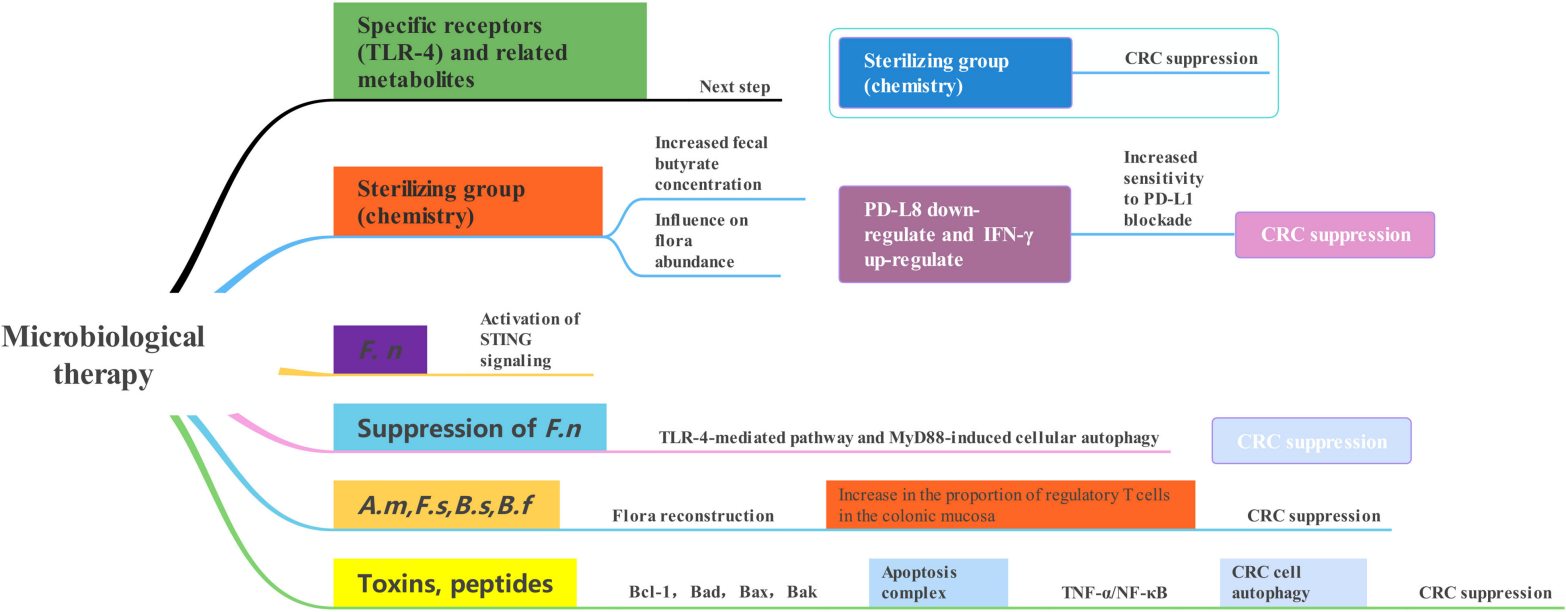
Microbiological therapy for colorectal cancer.

**Figure 3:**
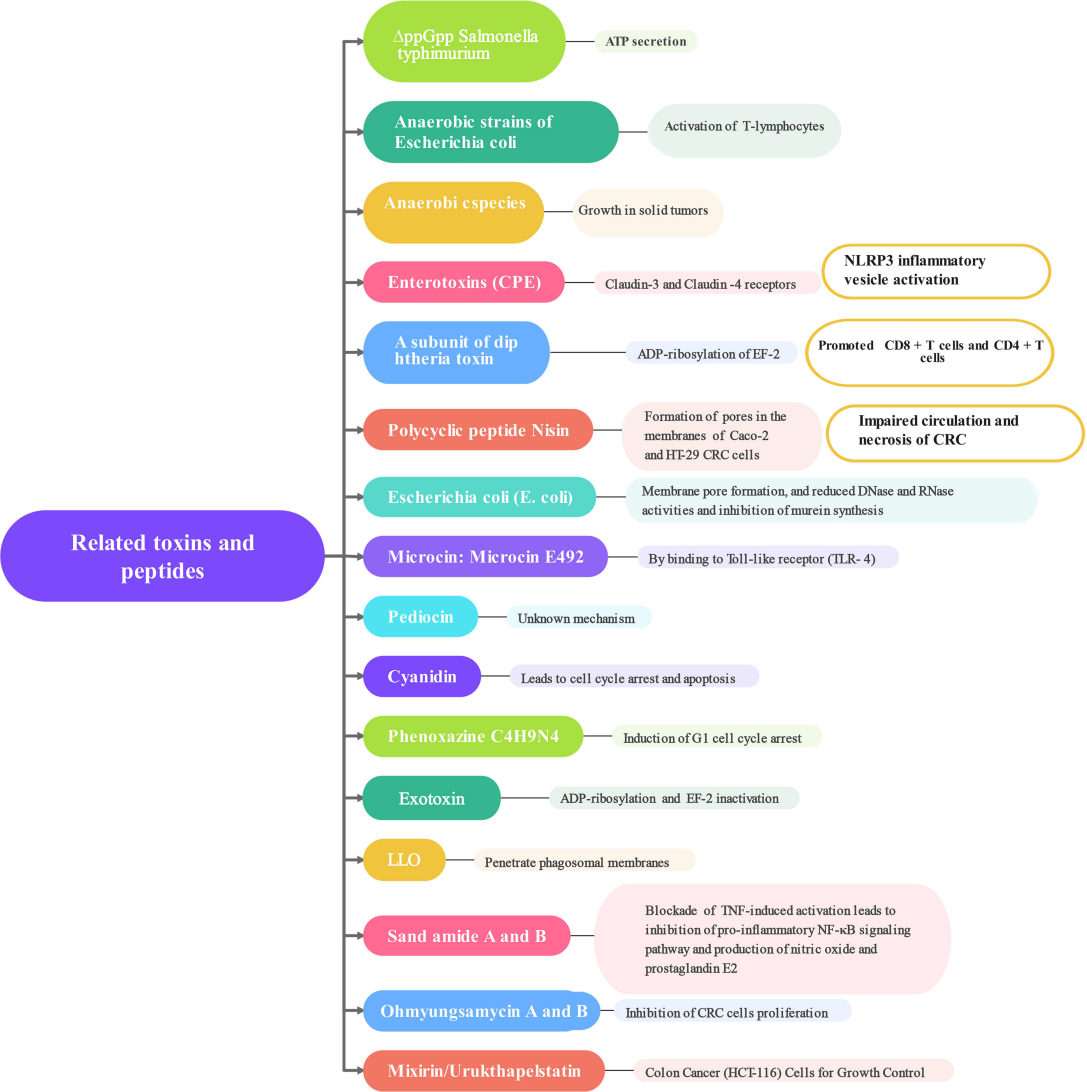
Toxins and peptides related to microbiological therapy.

The connection between CRC and the gut microbiota is strong. While we still don’t fully understand how the microbiota impacts the development and progression of CRC, there is increasing proof that it plays a direct role in influencing signaling pathways, anti-tumor immune responses, and cell growth (Montalban-Arques and Scharl, 2019). It has been shown that the gut microbiota immune system kills the bacterial flora through specific receptors (Toll-like receptors) and related metabolites (**Figure 2**). *Clostridium nucleatum, Escherichia coli*, and *Mimicronium fragilis* play a crucial role in the development of CRC. Increasing dietary fiber, including fructans and oligogalactans, has an inhibitory effect on CRC, but it also affects the abundance of Bifidobacteria and Lactobacillus, which increases fecal butyrate concentrations (Rebersek, 2021). It has been reported that intestinal flora plays an anti-cancer role in the efficacy of PD-L1 immune checkpoint inhibitor blockade (Yu, 2018). *F. nucleatum* has been shown to induce different immune responses in CRCs with varying microsatellite instability (MSI) states. *F. nucleatum* could induce PD-L8 expression by activating STING signaling during PD-L1 blockade therapy and increase the interferon-gamma (IFN-γ) CD1 tumor-infiltrating lymphocytes (TILs), which increases tumor sensitivity to PD-L1 blockade (Gao et al., 2021). It has also been reported that inhibition of *F. nucleatum* and reduction of its abundance modulate the TLR-4-mediated pathway and MyD88-induced cellular autophagy, which may enhance the chemotherapeutic effect of CRC (Mima et al., 2015; Yu et al., 2017). Simultaneously, the restoration of the gut microbiota composition can lead to the augmentation of regulatory T cell populations within the colonic mucosa (Routy et al., 2018; Shi et al., 2023). According to recent studies, the anticancer effects of microbial therapies such as bacterial therapies are mainly manifested in the form of bacterial-related biologics, including toxins and peptides (Mueller et al., 2022). These compounds produce regulatory cytokines, like TNF-α, which leads to the activation or blocking of NF-κB, and they also activate pro-apoptotic proteins (Bcl-1, Bad, Bax, Bak), combine cytochrome C with caspase-9 to form an apoptotic complex, and ultimately promote CRC cells apoptosis. Apoptosis is a key target of cancer therapy and is characterized by an imbalance between cell proliferation and death, resulting in autophagy in CRC cells (Mueller et al., 2022). Next, how the following related strains and their metabolites combat CRC was explored (**Figure 3**). According to some studies, timulation of the inflammatory vesicle pathway triggered by bacteria can activate the immune system, and Δ*ppGpp Salmonella typhimurium* inhibits primary and even metastatic CRC by secreting ATP, which causes activation of the NLRP3 inflammatory vesicle in macrophages (Mengesha et al., 2007; Nguyen et al., 2010). It has also been shown that the anaerobic strain of *E. coli* counteracts CRC cells by activating the production of T-lymphocytes, thereby greatly contributing to the tumor-protective activity of CD8+ and CD4+ T-cells (Azadi et al., 2021). At the same time, anaerobic bacterial species can invade and grow in solid tumors, allowing impaired circulation and necrosis of CRC (Fox et al., 1996; Zhao et al., 2005; Agrawal et al., 2017; Kasper et al., 2020). The antagonistic effect of related toxins on CRC was also investigated. Based on relevant reports and experiments, it has been shown that Clostridium perfringens enterotoxin (CPE) produced by *Clostridium perfringens* can bind to Claudin-3 and -4 receptors on the surface of CRC, leading to the breakdown of cellular osmotic homeostasis and the lysis of cancer cells (Pahle et al., 2017; Sasaki et al., 2020). The subunit derived from Gram-positive Corynebacterium diphtheriae can halt protein production by ADP-ribosylating cytoplasmic elongation factor 2 (EF-2), eventually resulting in the demise of CRC cells (Vallera et al., 2002; Martarelli et al., 2009). The polycyclic peptide Nisin secreted by *Lactococcus lactis* strains enables the formation of pores in the membranes of Caco-2 and HT-29 CRC cells ultimately leading to membrane depolarization and apoptosis in CRC cells (Ahmadi et al., 2017). Cytotoxic effects of colistin on CRC cells include membrane pore formation, reduced DNase and RNase activities, and inhibition of murein synthesis (Kohoutova et al., 2020). Microcin/Microcin E492 causes apoptosis by enabling pore formation in CRC cell membranes and ultimately by binding to Toll-like receptor 4 (Hetz et al., 2002; Lagos et al., 2009). Pediocin has been observed to trigger apoptosis through a mechanism that remains unidentified (Mueller et al., 2022). Proteins capable of entering CRC cells and inducing cell cycle arrest and apoptosis by aspyrins (Mueller et al., 2022). Phenazine, a nitrogen-containing metabolite, is produced by various bacterial strains, with notable secretion observed in numerous Pseudomonas aeruginosa strains. This compound includes phenazine 1-carboxylic acid and phenazine 1,6-dicarboxylic acid (PDC) (Wolf and Elsässer-Beile, 2009). Crucially, it induces G1 cell-cycle arrest, consequently prompting apoptosis, while also negatively impacting CRC cell viability and hampering DNA synthesis (Iglewski and Kabat, 1975; Wolf and Elsässer-Beile, 2009). Recall antigens delivered via Listeria might serve as a viable option for cancer immunotherapy beyond neoantigens (Selvanesan et al., 2022). Listeriolysin O (LLO), a poisonous compound produced by the anaerobic microorganism Listeria monocytogenes, possesses the ability to infiltrate the cytoplasm of antigen-presenting cells and rupture the phagosome membranes (Mueller et al., 2022).

Following that, non-ribosomal peptides are discussed, which constitute an alternative group of peptides produced by bacteria, fungi, and cyanobacteria. These peptides play a role in combatting CRC. Lucentamycins, Arenamides, Ohmyungsamycins, Mixirins, and Urukthapelstatin A possess the ability to engage with CRC cells, either through direct interactions or indirect mechanisms (Sacks et al., 2018). For instance, sarcosamides A and B have demonstrated their potential in inhibiting the pro-inflammatory NF-κB signaling pathway by effectively blocking TNF-induced activation, ultimately leading to a reduction in inflammation (Byun et al., 2020). Consequently, this decrease in inflammation hinders the production of NO and PGE2, effectively opposing the activities of CRC cells (Byun et al., 2020). Cyclic depsipeptides, specifically Ohmyungsamycin A and B, display a discerning ability to impede the proliferation of CRC cells (Um et al., 2013; Byun et al., 2020). Mixirin, derived from *Bacillus marinus*, is a cyclic thiopeptide that can exhibit cytotoxicity against the HCT-116 (human colon cancer cell line) (Yamamoto et al., 2015). Urukthapelstatin A is a cyclic sulfur peptide produced by *Mechercharimyces asporophorigenens*, a marine microorganism affiliated with the *Thermoactinobacteriaceae* family (Matsuo et al., 2007). This compound exerts inhibitory effects on the proliferation of HCT-116 cell line through its biological activity (Mueller et al., 2022).

## 7 Conclusion

Intestinal microorganisms constitute a rich ecosystem, with more than 1000 species of bacteria belonging to 50 genera and 17 families. Their composition depends largely on environmental conditions, and there are differences among individuals. With the in-depth study of intestinal bacteria, we can find that intestinal bacteria and their metabolites have many effects on CRC, such as inflammatory transformation, malignant transformation of intestinal polyps, tumor escape, treatment and so on. According to relevant studies, it can be reported that apoptosis of CRC cells can be induced by inhibiting the activity of glutamate dehydrogenase, regulating the MAPK signaling pathway, PI3K/AKT, and other related pathway mechanisms, which are crucial for the development of CRC (Chang and Kang, 2023; Yang et al., 2023).

In this paper, we reviewed that intestinal bacteria can participate in adenoma-adenocarcinoma transformation through their metabolites and affect the DNA coding of intestinal cells. It is believed that in the initial stage of CRC, “driver” bacteria are dominant in the intestine, which leads to adenoma and even malignant tumor with the increase of DNA damage and chromosome instability in intestinal cells. In addition, intestinal flora can directly induce tumor-associated immune cell infiltration and promote the formation of tumor microenvironment. In some familial hereditary adenomatous polyposis, specific intestinal bacteria often play a role in promoting the carcinogenesis of adenomas. No matter which kind of colon cancer patients, the determination of intestinal flora and its metabolites has great clinical significance, because it may early warn the occurrence of colorectal cancer and adenoma, or improve the prognosis of patients with CRC. Tailoring the regulation of gut microbiota on an individual basis is poised to emerge as a focal point and innovative strategy in the realm of preventing and supporting the treatment of CRC.

## Author contributions

JH: Conceptualization, Writing – original draft, Writing – review & editing. BZ: Conceptualization, Writing – original draft, Writing – review & editing. YZ: Conceptualization, Writing – original draft, Writing – review & editing. TY: Conceptualization, Writing – original draft, Writing – review & editing. YC: Conceptualization, Writing – original draft, Writing – review & editing. JL: Conceptualization, Writing – original draft, Writing – review & editing. YY: Conceptualization, Writing – original draft, Writing – review & editing. HS: Conceptualization, Writing – original draft, Writing – review & editing. DS: Conceptualization, Writing – original draft, Writing – review & editing.
